# Biosynthesis of β-nicotinamide mononucleotide from glucose via a new pathway in *Bacillus subtilis*

**DOI:** 10.3389/fmicb.2024.1405736

**Published:** 2024-06-11

**Authors:** Zhilei Tan, Yihang Yang, Yannan Wu, Jiajia Yan, Bin Zhang, Ying Hou, Shiru Jia

**Affiliations:** State Key Laboratory of Food Nutrition and Safety, Key Laboratory of Industrial Fermentation Microbiology, Ministry of Education, Tianjin University of Science and Technology, Tianjin, China

**Keywords:** β-nicotinamide mononucleotide, *Bacillus subtilis*, metabolic engineering, microbial synthesis, fermentation

## Abstract

**Introduction:**

β-nicotinamide mononucleotide (β-NMN) is an essential precursor of nicotinamide adenine dinucleotide (NAD^+^) and plays a key role in supplying NAD^+^ and maintaining its levels. Existing methods for NMN production have some limitations, including low substrate availability, complex synthetic routes, and low synthetic efficiency, which result in low titers and high costs.

**Methods:**

We constructed high-titer, genetically engineered strains that produce NMN through a new pathway. *Bacillus subtilis* WB600 was used as a safe chassis strain. Multiple strains overexpressing *NadE*, *PncB*, and *PnuC* in various combinations were constructed, and NMN titers of different strains were compared via shake-flask culture.

**Results:**

The results revealed that the strain *B. subtilis PncB1-PnuC* exhibited the highest total and extracellular NMN titers. Subsequently, the engineered strains were cultured in a 5-L fermenter using batch and fed-batch fermentation. *B. subtilis PncB1-PnuC* achieved an NMN titer of 3,398 mg/L via fed-batch fermentation and glucose supplementation, which was 30.72% higher than that achieved via batch fermentation.

**Discussion:**

This study provides a safe and economical approach for producing NMN on an industrial scale.

## 1 Introduction

β-nicotinamide mononucleotide (β-NMN) is a bioactive substance with essential functions in the human body. NMN can be converted into nicotinamide adenine dinucleotide (NAD^+^), which is a coenzyme that participates in NAD-dependent signal transduction and acts as an electron carrier for metabolic redox reactions. When NAD^+^ is deficient, supplementation with additional NMN can increase the NAD^+^ content in the body to prevent Parkinson’s disease (Lu et al., 2014; Martin et al., 2017), regulate metabolism, reduce apoptosis, and maintain the redox status (Alano et al., 2004). Moreover, NMN supplementation can prevent DNA damage and accumulation of reactive oxygen species (ROS) (Tarantini et al., 2019). Furthermore, NMN exerts neuroprotective effects and improves cognitive and behavioral functions (Li et al., 2017; Johnson et al., 2018; Hosseini et al., 2019; Miao et al., 2020). Recent studies have reported that NMN supplementation exerts therapeutic effects on chronic inflammation and retinal damage, promotes melanogenesis (Chen et al., 2020; Liu et al., 2021; Lin et al., 2021b; Brito et al., 2022), and helps prevent skin photoaging, glaucoma, and cisplatin-induced ototoxicity (Katayoshi et al., 2021; Petriti et al., 2021; Zhan et al., 2021).

**Figure 1:**
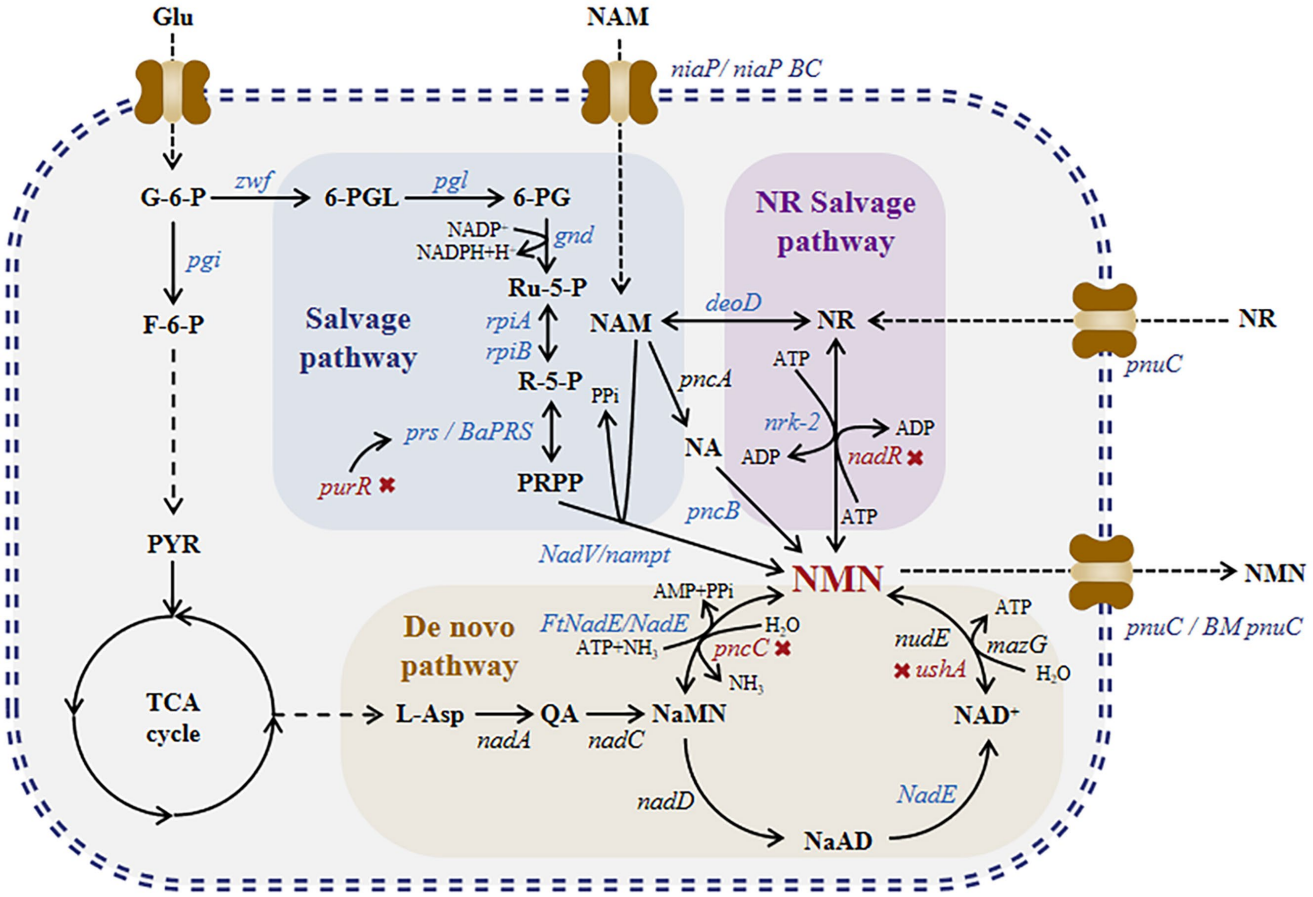
NMN biosynthesis pathway. Nicotinamide mononucleotide synthesis: *de novo*, NR salvage, and salvage routes. NaMN is produced through the *de novo* synthesis of NMN, which is catalyzed via NADA and NADC, leading to the production of NAD^+^. NAD^+^ can be broken down into NMN via nudix hydrolases, such as NUDE (nudE) or other nucleotidases (*mazG*), with comparable hydrolytic activity. Alternatively, NaMN may be directly converted into NMN by NMN synthetase. For the NR salvage route, nicotinamide riboside kinase (*nrk*) transforms NR into NMN. Within the salvage process, NAM phosphoribosyl transferase (*nampt*) transforms NAM into NMN. Abbreviations of NMN intermediates are shown in standard red bold font. Asp., iminoaspartate; QA, quinolinic acid; NaMN, nicotinic acid mononucleotide; AMP, adenosine monophosphate; NA, nicotinic acid; NaAD, deamido-nicotinamide adenine dinucleotide; NAD^+^, nicotinamide adenine dinucleotide; NMN, nicotinamide mononucleotide; NR, nicotinamide riboside; ATP, adenosine triphosphate; ADP, adenosine diphosphate; NAM, nicotinamide; PRPP, 50-phosphoribosyl1-pyrophosphate; PPi, pyrophosphate; Pi, phosphate; NADP^+^, nicotinamide adenine dinucleotide phosphate. Abbreviations of metabolites: Glu, glucose; G-6-P, glucose-6-phosphate; F-6-P, fructose-6-phosphate; PYR, pyruvic acid; 6-PGL, 6-phosphogluconolactone; 6-PG, 6-phosphogluconic acid; Ru-5-P, ribulose 5-phosphate; and R-5-P, ribulose 5-phosphate. The genes and enzymes associated with the reactions include the following: *nadA*, quinolinate synthase; *nadC*, quinolinate phosphoribosyltransferase; *nadD*, NaMN adenylyltransferase; *FtNadE*, NMN synthase from *Francisella tularensis*; *nudE*, NADH hydrolase; *mazG*, nucleoside triphosphate pyrophosphohy-drolase; *ushA*, UDP-sugar hydrolase; *pncC*, NMN amidohydrolase; *pncB*, nicotinic acid phosphate ribose transferase; *nadR*, NMN amidohydrolase from *B. subtilis*; nrk-2, human nicotinamide riboside kinase 2; *deoD*, purine-nucleoside phosphorylase; *prs*, ribose-phosphate diphosphokinase; *BaPRS*, ribose-phosphate diphosphokinase from *Bacillus amyloliquefaciens*; *purR*, Pur operon repressor; *nadV*, nicotinamide phosphoribosyltransferase; nampt, nicotinamide phosphoribosyltransferase; *rpiA*, ribose 5-phosphate isomerase A; *rpiB*, ribose 5-phosphate isomerase B; *gnd*, 6-phosphogluconate dehydrogenase; *pgl*, 6-phosphogluconolactonase; *zwf*, glucose 6-phosphate dehydrogenase; and *pgi*, glucose-6-phosphate isomerase. Four transporters, namely, *pnuC* (nicotinamide riboside transporter), *BMpnuC* (nicotinamide riboside transporter from *Bacillus mycoides*), *niaP* (niacin transporter), and *niaPBC* (nicotinamide transporter), are shown next to the transporter protein icon.

Currently, NMN is synthesized via chemical, enzymatic, and biosynthetic methods. NMN synthesis via chemical methods such as ketalization protection using nicotinamide ribose as a substrate involves a three-step process-protection of ketalization reagent, phosphorylation, and deprotection (Sauve and Mohammed, 2021). Although these methods produce high amounts of NMN, they are expensive. NMN synthesis via enzymatic methods mimics the reaction pathways of NAD^+^ and NMN in organisms. In 1957, Jack et al. successfully performed the first enzymatic synthesis of NMN (Preiss and Handler, 1957). They synthesized NMN using nicotinamide (NAM) as a substrate in the presence of nicotinamide phosphoribosyltransferase (NAMPT) and phosphoribosylpyrophosphate synthetases (PRPPs). Fu et al. developed a method to produce NMN from nicotinamide (NAM), ribose, and ATP under the action of nicotinamide phosphoribosyltransferase, ribose-phosphate pyrophosphate kinase, and ribokinase (Fu and Zhang, 2018). In 2018, Zhu et al. modified this method and increased the substrate conversion rate to 99.5% (Wei and Zhang, 2018). Qian et al. conducted a study on the nicotinamide riboside (NR) salvage pathway and identified the enzyme nicotinamide riboside kinase in *Kluyveromyces marxianus* that can convert NR into NMN, with a titer of 281 g/[L day] (Qian et al., 2022). He et al. added human-derived NRK2 to the surface of *Saccharomyces cerevisiae* cells and produced NMN from NR with a titer of 12.6 g/L (He et al., 2022). However, owing to the high cost of enzyme purification required for enzymatic synthesis, fermentation and whole-cell catalysis methods have been recently developed for NMN production. NMN biosynthesis can be achieved via two routes (Figure 1): the salvage pathway and *de novo* pathway. Marinescu et al. conducted a study on these two pathways; they overexpressed *HdNadV* in *Escherichia coli*, which resulted in a significant increase in NMN titer of up to 15.42 mg/L via NAM supplementation (Marinescu et al., 2018). Shoji et al. discovered and overexpressed *NiaP*, *PnuC*, and *NAMPT* in *Bacillus cereus*, which markedly increased the NMN titer to 6.79 g/L via whole-cell catalysis with NAM supplementation (Shoji et al., 2021). As shown in Figure 1, nicotinic acid mononucleotide (NAMN) can be converted into NMN through ammonia-dependent NAD^+^ synthetase (FtNadE). Black et al. increased the intracellular accumulation of NMN by 1,000-fold (501 mg/L) by overexpressing *FtNadE* and knocking out *pncC* and *nadR* in *E. coli* (Black et al., 2020). Huang et al. used a *BaPRS*-*VpNadV*-*BmPnuC*-overexpressing *E. coli* strain, which produced NMN with a titer of 16.2 g/L via fed-batch fermentation and NAM supplementation (Huang et al., 2022).

**Figure 2:**
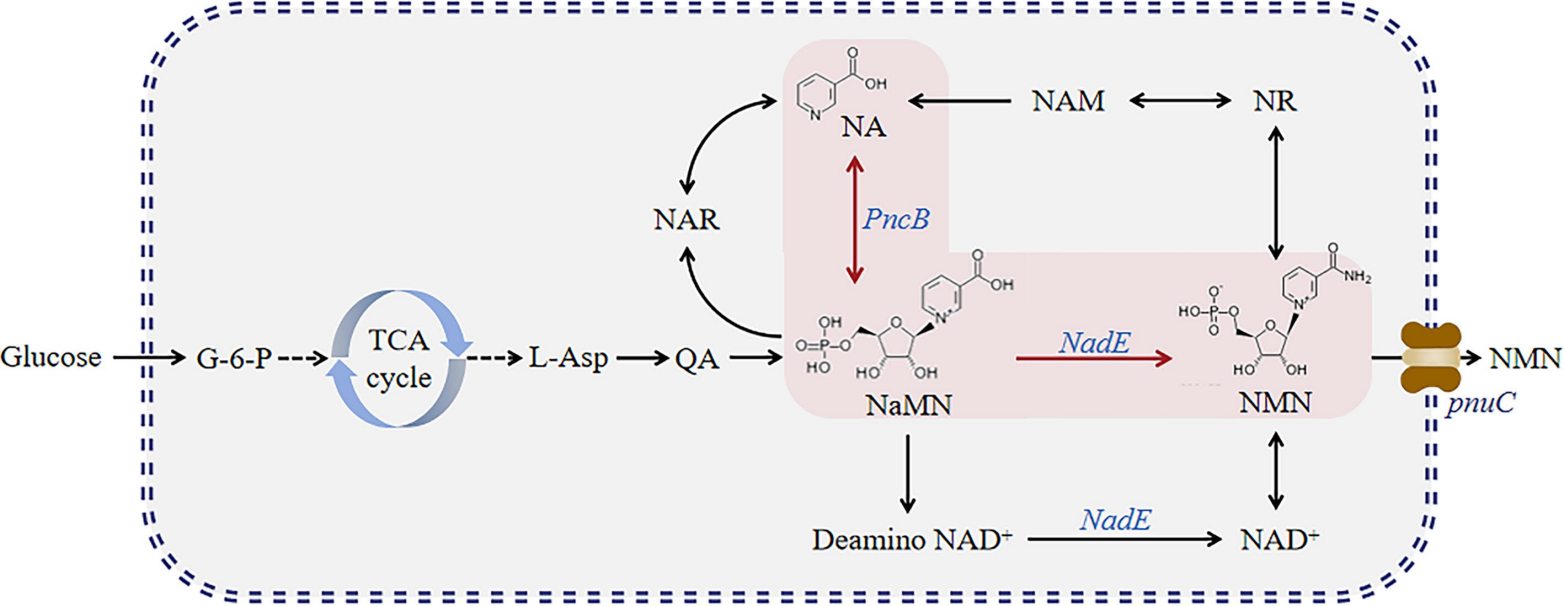
NMN synthesis pathway developed in this study. Glucose enters the tryptophan pathway via glycolysis to produce quinolinic acid and further forms NMN via two pathways.

This process may become more complex and expensive when additional substrates, such as NR and NAM, are added. Therefore, we attempted to construct a different pathway (Figure 2). Herein, we established six genetically engineered strains and determined their NMN titers. First, we overexpressed *PncB* and *NadE* in *B. subtilis*. Then, *PnuC* in *B. mycides* was also overexpressed to enable the transportation of NMN in *B. subtilis*. We found that the constructed strains can utilize glucose to synthesize NMN directly.

## 2 Materials and methods

### 2.1 Chemicals and reagents

The target gene *PnuC* (gene ID: 66264729) was synthesized by Universe Gene Technology (Tianjin) Co., Ltd. (Tianjin, China), and its sequence was codon-optimized for expression in *B. subtilis*. Oligonucleotide primers were synthesized by GENEWIZ Bio, Inc. (Suzhou, China). NMN was purchased from Shanghai Bide Pharmaceutical Technology Co., Ltd. (Shanghai, China) and used for high-performance liquid chromatography (HPLC) analysis. Unless otherwise specified, all organic reagents were purchased from Beijing AoBoXing Bio-Technology Co. Ltd. (Beijing, China), and all inorganic reagents were purchased from China National Medicines Corporation Ltd. (Beijing, China).

### 2.2 Strain construction

The strains and plasmids used in this study are listed in Supplementary Table S1. The protease-deficient strain *B. subtilis* WB600 (Wu et al., 1991) and pMA5 (Zhu et al., 2020) were used as the host strain and overexpressing vector, respectively. Compared with integrated plasmids, the shuttle plasmid PMA5 has the advantage of having a higher copy number and a stronger *HpaII* promoter. In addition to *Bacillus mycoides PnuC*, *B. subtilis* 168 was used as a source of genomic DNA for amplifying NMN biosynthesis genes. All primers listed in Supplementary Table S2 were designed based on the genomic sequences of *B. subtilis* 168.

**Figure 3:**
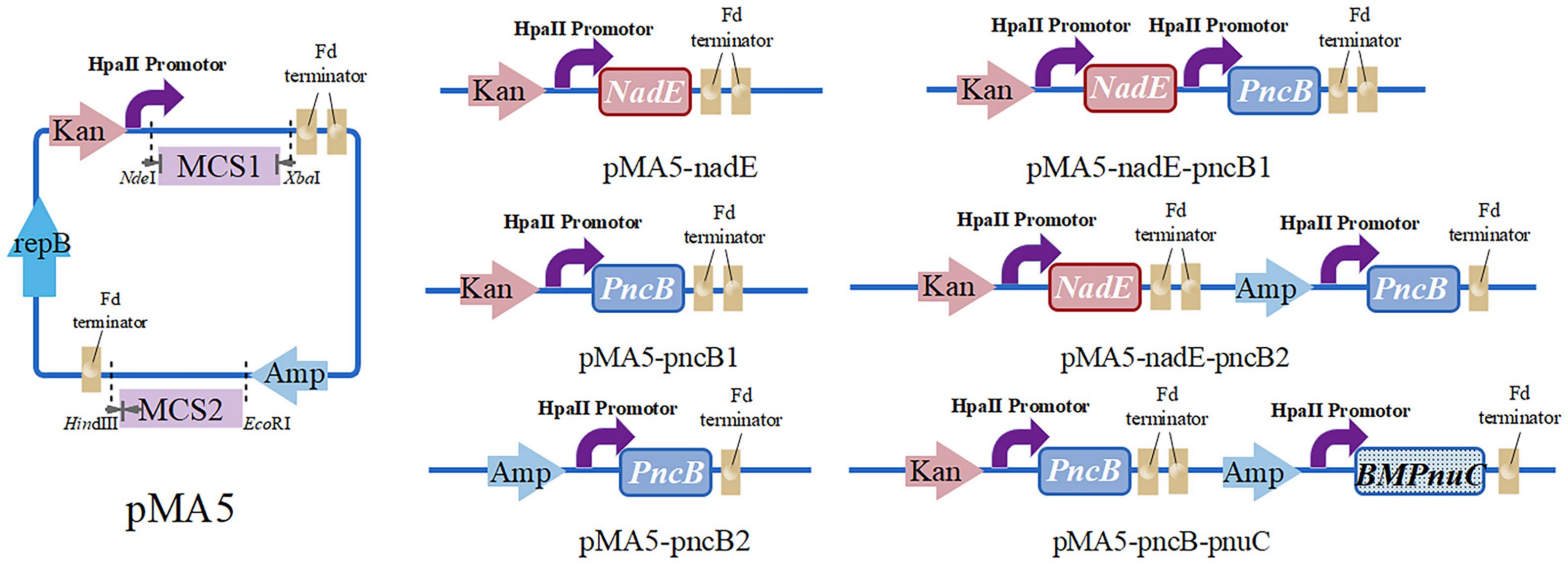
Plasmid constructs for NMN production, including pMA5-nadE, pMA5-pncB1, pMA5-pncB2, pMA5-nadE-pncB1, pMA5-nadE-pncB2, and pMA5-pncB-pnuC.

To construct the recombinant plasmid pMA5-nadE, the primers B. N-F/R were used to amplify *NadE* gene from *B. subtilis* 168 chromosomal DNA, and the resulting fragment was cloned into *Nde*I/*Bam*HI-digested pMA5 at multiple cloning site 1 (MCS1) using ClonExpress II One Step Cloning Kit (Nanjing Vazyme Biotech Co., Ltd., Nanjing, China), according to the manufacturer’s instructions. The recombinant plasmid pMA5-pncB1 was constructed as follows. The entire fragment of *PncB* (amplified using primers B. P1-F/R) was purified and cloned into *Nde*I-digested pMA5 at MCS1. The recombinant plasmid pMA5-pncB2 was constructed using *PncB* as mentioned above and then cloned into *Hind*III-digested pMA5 at MCS2. To construct a multigene coexpression plasmid, different enzyme cleavage sites were selected based on the sequence of the single-gene expression plasmids. *NadE* and *PncB* (with *Bam*HI and *Hind*III restriction sites) were inserted at MCS1 and MCS2, respectively. To generate pMA5-nadE-pncB1 plasmid, pMA5 was linearized using *Bam*HI and *Nde*I, and the amplification products of *NadE* and *PncB* were inserted at MCS1. The recombinant plasmid pMA5-nadE-pncB2 was constructed by inserting *PncB* at MCS2 of *Hin*dIII-digested pMA5-nadE. The *B. mycoides PnuC* sequence was synthesized by Universe Gene Technology Co., Ltd. (Tianjin) and cloned into the recombinant plasmid pMA5-pncB1 (*Hin*dIII-digested) at MCS2 to establish the recombinant vector pMA5-pncB-pnuC. The resulting plasmids (Figure 3) were used for the transformation of *B. subtilis* WB600 (Anagnostopoulos and Spizizen, 1961). For selection, all transformants were plated onto Luria broth (LB) agar plates containing kanamycin (100 μg/mL), and positive transformants were verified via colony PCR.

### 2.3 Medium and culture conditions

*Bacillus subtilis* strains were routinely cultured at 37°C in LB medium (consisting of 10 g/L tryptone, 5 g/L yeast extract, and 10 g/L NaCl) or on LB plates containing 1.5–2% (w/v) agar supplemented with appropriate antibiotics as necessary (kanamycin, 50–100 μg/mL). All recombinant *B. subtilis* cells precultured overnight at 37°C in LB medium containing kanamycin (50 μg/mL) under shaking conditions at 220 rpm were used as seed cultures.

For flask cultivation, the seed culture (5%) was inoculated into 500 mL baffled flasks containing 50 mL of liquid medium (12 g/L tryptone, 24 g/L yeast extract, 12.53 g/L K_2_HPO_4_, 2.31 g/L KH_2_PO_4_, and 20 g/L glucose) supplemented with kanamycin (50 μg/mL). The cultures were incubated at 37°C and 220 rpm for approximately 24 h.

Batch fermentation was performed in a 5-L bioreactor (5BG, Baoxing, China). The seed culture (5%) was inoculated into a 2-L liquid medium. The culture temperature was maintained at 37°C, and the initial pH was set at approximately 7.0. The airflow rate was set at 1 L/min, and the dissolved oxygen (DO) content was maintained at 30% by automatically varying the agitation speed (400–800 rpm). During the initial stage of fermentation, the OD_600_ of the fermentation broth was similar to that of the blank culture medium, i.e., close to zero.

The inoculation amount, liquid medium composition, and DO content for fed-batch fermentation in a 5-L fermenter were identical to those for batch fermentation. The pH was automatically maintained at approximately 6.5 using 2 M HCl and NaOH solution after fermentation for 4 h. The residual glucose (RG) content was maintained at 10 g/L by adding a glucose solution (400 g/L) when the RG content was reduced to approximately 8 g/L. A biosensor (SBA-40C, Institute of Biology, Shandong Academy of Sciences) was used to quantitate RG content.

### 2.4 Quantitative reverse transcription-polymerase chain reaction

To confirm the relative transcriptional levels of *PncB*, *NadE*, and *PnuC*, we performed qRT-PCR. Total RNA was extracted from different *B. subtilis* strains cultured in a shake flask for 12 h using Bacterial RNA Kit (Omega Bio-Tek, Inc.). Reverse transcription was performed using PrimeScript^™^ RT Reagent Kit (Takara Bio Inc.). qRT-PCR was performed using the StepOnePlus Real-Time PCR System (Applied Biosystems, Inc.). The specific primers designed in this study are listed in Supplementary Table S3. The 16S rRNA gene encoding the major sigma factor in *B. subtilis* was used as the internal control (Klappenbach et al., 2000), and relative quantification was performed using the comparative cycle threshold method (Proctor et al., 2018).

### 2.5 HPLC analysis

To quantify extracellular and intracellular NMN, 1 mL of cultured cells was collected and centrifuged for 5 min at 4000 rpm and 4°C. The supernatant was used as the extracellular sample. After discarding the supernatant, the bacterial pellet was resuspended in 1 mL of lysozyme solution (4 mg/mL), and the mixture was incubated for 30 min at 37°C. Cell lysis was performed on ice via ultrasonication (400 W) for 30 cycles (each for 3 s with 2 s intervals), and the suspension was centrifuged for 5 min at 6000 rpm and 4°C. The supernatant was collected as the intracellular sample. To measure total NMN titer, lysozyme was added to the culture broth, and the mixture incubated for 30 min at 37°C. The whole-cell lysate was ultrasonicated and centrifuged as described above. The supernatants were collected and used as total NMN samples.

All NMN samples were filtered through a 0.22 μm PES membrane before analysis using an HPLC instrument (Agilent 1,260 Series) equipped with a TSKgel ODS-80TS quinolinic acid (QA) column (4.6 mm × 25 cm, 5 μm, Tosoh). The mobile phase comprised methanol and ion pair aqueous solution (5.4 g of KH₂PO₄ and 1.7 g of tetrabutylammonium hydrogen sulfate were dissolved in 1000 mL of water; pH 6.0) at a ratio of 30:70, with a flow rate of 1 mL/min. The eluates were monitored at 260 nm, and the column temperature was set at 28°C (Stocchi et al., 1987). NAM, nicotinic acid (NA), NR, NAD^+^ standard sample processing methods were the same as described above. The standard NMN and NAD^+^ samples were diluted to different concentrations, and a standard curve was constructed to calculate NMN and NAD^+^ contents.

## 3 Results

### 3.1 Establishment of novel NMN synthesis pathways in *Bacillus subtilis*

The NMN biosynthesis pathway primarily uses NAM or NR as a precursor (Figure 1), which requires high production costs. Nicotinic acid mononucleotide (NaMN) biosynthesis can be achieved via two main routes (Figure 2): (1) biosynthesis of NaMN from NA via nicotinic acid phosphate ribose transferase (PncB); (2) conversion of QA, which is biosynthesized from glucose through the TCA cycle, into NaMN. NMN synthesis via esterification of NA to NaMN and conversion of the hydroxyl group of NaMN to an amino group is a pathway for NMN production in *B. subtilis*.

**Figure 4:**
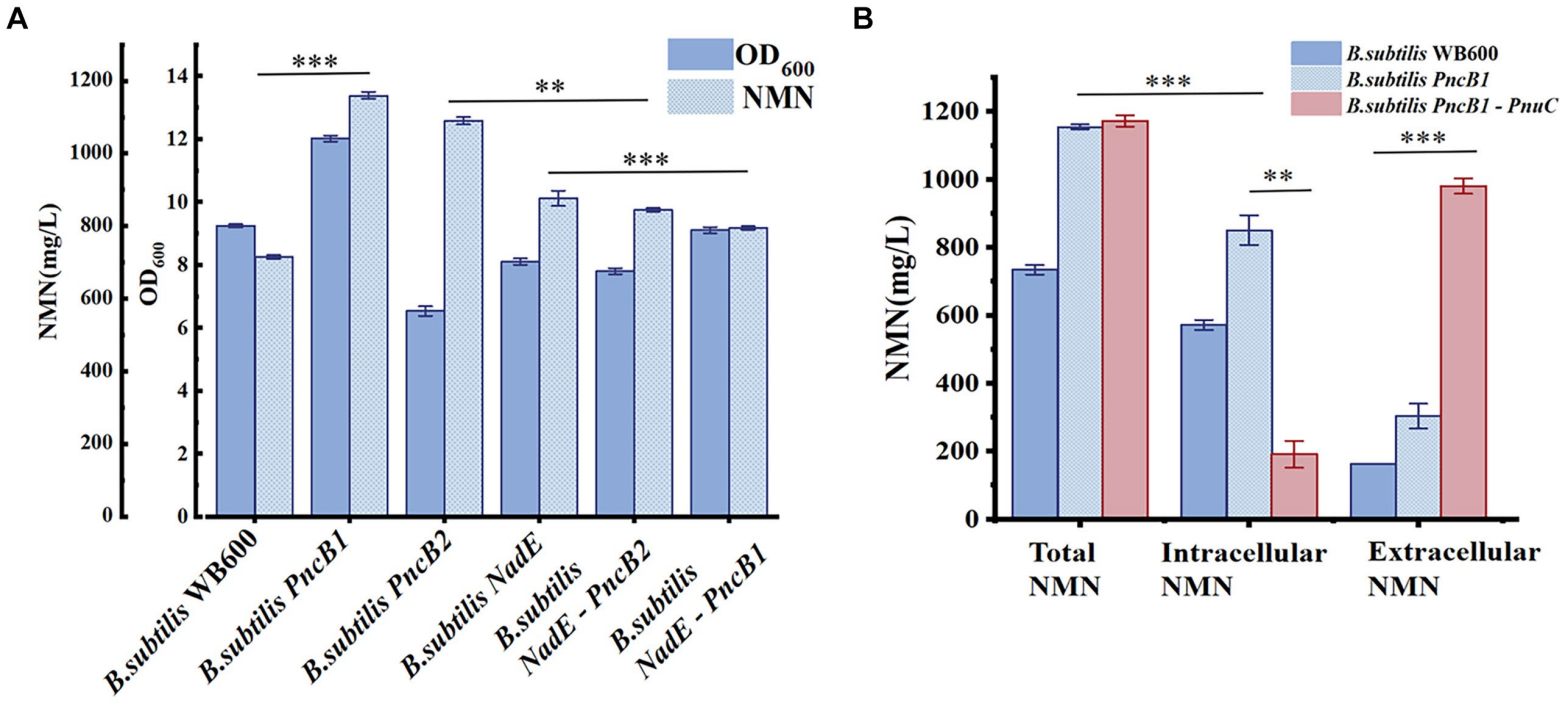
**(A)** Biomass (OD_600_) and NMN production of different *B. subtilis* strains during flask fermentation. **(B)** Effect of transporter protein addition on NMN production by genetically engineered *B. subtilis* strains. The mean and standard deviation of three independent experiments with biological replicates are shown using the values and error bars. Using a two-tailed *t*-test, *p < 0.05, **p < 0.01, and ***p < 0.001.

*PncB* can increase the NaMN production rate (Wünsche et al., 2012), thereby promoting NMN biosynthesis. *NadE* catalyzes the direct synthesis of NMN from NaMN and participates in the NAD^+^ salvage pathway to further enhance NMN production. To decrease production costs, we increased the expression of *PncB* in *B. subtilis* WB600 and used glucose as a substrate to produce NMN without adding expensive substrates. The pMA5 plasmid contains two MCSs (MCS1 and MCS2). To compare the expression effects of MCS1 and MCS2, we constructed recombinant plasmids pMA5-PncB1 and pMA5-PncB2 (Figure 3), which were subsequently transformed into *B. subtilis* WB600, resulting in the generation of two genetically engineered strains *B. subtilis PncB1* and *B. subtilis PncB2*. After culture for 12 h in the shake flask, the strains *B. subtilis* WB600, *B. subtilis PncB1*, and *B. subtilis PncB2* produced 720.05, 1160.80, and 1097.10 mg/L NMN, respectively (Figure 4A). Subsequently, we further quantified the NAD^+^ contents of strains *B. subtilis PncB1* and *B. subtilis* WB600 (Supplementary Figure S1). After shake-flask fermentation, the NAD^+^ content of the strain *B. subtilis PncB1* (265.41 mg/L) at 12 h was 37.2% higher than that of the control strain WB600 (193.46 mg/L). These results indicate that *PncB* overexpression positively impacts the production of NMN and NAD^+^ by these engineered strains, and MCS1 appears to have a superior overexpression effect compared with MCS2.

As shown in Figure 1, the *de novo* synthesis of NMN is mediated by *nadD* and *NadE*, leading to the production of NAD^+^. After NAD^+^ generation through this pathway, *nudE* can degrade NAD^+^ into NMN (Rodionov et al., 2008; Osterman, 2009), and *NadE* participates in the direct conversion of NaMN to NMN. Sorci et al. revealed that NadE encoded by *FtNadE* can directly catalyze the conversion of NaMN into NMN in *Francisella tularensis* (Sorci et al., 2009), without the need to participate in the NAD^+^ remedial synthesis pathway. To enhance NMN biosynthesis, in addition to overexpressing *PncB* in *B. subtilis* to increase the conversion of NaMN to NMN, we attempted to overexpress *NadE* in *B. subtilis* (Figure 2). The recombinant plasmid pMA5-NadE was constructed (Figure 3) to overexpress *NadE*. This plasmid was transformed into *B. subtilis* WB600 to obtain the engineered strain *B. subtilis NadE*. The NMN titer of *B. subtilis NadE* reached approximately 969.69 mg/L after 12 h of fermentation in the flask, which was 34.67% higher than that of the host strain *B. subtilis* WB600. NadE and PncB were inserted at different MCSs of the plasmid pMA5 and overexpressed in the host strain *B. subtilis* WB600, resulting in the generation of strains *B. subtilis NadE-PncB1* and *B. subtilis NadE-PncB2*, which produced 821.05 and 941.38 mg/L NMN, respectively. However, compared with the individual overexpression of *PncB* or *NadE*, their combined overexpression did not enhance the NMN titer (Figure 4A). The shake-flask culture results indicated that genetically engineered strains increased their NMN titers by 16–64% compared with the host strain *B. subtilis* WB600 (Figure 4A).

### 3.2 Further strain optimization via *PnuC* expression

There are several pathways for the synthesis and recycling of NMN in *B. subtilis* cells, and intracellular NMN is utilized for bacterial growth (Magni et al., 1999). To increase the NMN titer, in addition to enhancing the expression of key enzymes in the NMN synthesis pathway, it is necessary to transport intracellular NMN to the extracellular environment to avoid degradation. Studies on transporter proteins that transport NMN to the extracellular environment (Grose et al., 2005; Shoji et al., 2021) have shown that overexpression of *PnuC* from *Bacillus mycoides* can efficiently increase the NMN titer (Sauer et al., 2004; Rodionov et al., 2008). As shown in Figure 4A, *B. subtilis* PncB1 exhibited the highest titer among the five engineered strains, with a 64% increase in titer relative to that in *B. subtilis* WB600. Therefore, *PnuC* was inserted into pMA5-PncB1 to generate the plasmid pMA5-PncB1-PnuC (Figure 3). In a study on transporter proteins, the control group comprised the strains *B. subtilis* WB600 and *B. subtilis PncB1*, whereas the experimental group contained the strain *B. subtilis PncB1*-*PnuC*. *B. subtilis* WB600, *B. subtilis PncB1*, and *B. subtilis PncB1-PnuC* were cultured under the same conditions. The titers of total NMN, extracellular NMN, and intracellular NMN produced by different strains were determined. As shown in Figure 4B, the total NMN titers of *B. subtilis PncB1* and *B. subtilis PncB1*-*PnuC* were significantly higher than the total NMN titer of *B. subtilis* WB600. However, *B. subtilis PncB1*-*PnuC* (1160.24 mg/L) did not show a considerable increase in total NMN titer compared with *B. subtilis PncB1* (1160.80 mg/L). Further analysis revealed that extracellular NMN accumulation of *B. subtilis PncB*-*PnuC* accounted for 83.69% of the total NMN titer. The extracellular NMN titers of *B. subtilis* WB600 and *B. subtilis PncB1* reached only 22.17 and 26.32% of the total NMN titer, respectively. At the time point of the highest cumulative titer, the extracellular NMN titer of *B. subtilis PncB1-PnuC* was significantly higher than the corresponding intracellular titer. In contrast, the extracellular NMN titers of *B. subtilis* WB600 and *B. subtilis PncB1* were significantly lower than the corresponding intracellular titers. To further verify that NMN was produced through strain fermentation, we measured the contents of NMN, NA, NAM, and NR in the blank culture medium. The results indicated that the culture medium contained trace amounts of NA (<29.7 mg/L) and NAM (<160 mg/L), further confirming that the engineered strains can utilize glucose to biosynthesize NMN (Supplementary Figure S2).

Taken together, the results indicate that *PnuC* is extremely effective in transporting intracellular NMN to the extracellular environment. *PnuC*-mediated excretion of NMN reduced intracellular NMN titers, thereby accelerating the conversion of NAM to NMN and preventing the undesired conversion of NMN to other compounds. Furthermore, even without exogenous NMN transporter proteins, a small amount of NMN accumulation was detected in the extracellular area of *B. subtilis*. However, similar results have not yet been reported for endogenous NMN transporters. The actual transmembrane transport process of wild-type bacterial NMN should be experimentally verified.

### 3.3 Characterization of gene expression in the engineered strains

**Figure 5:**
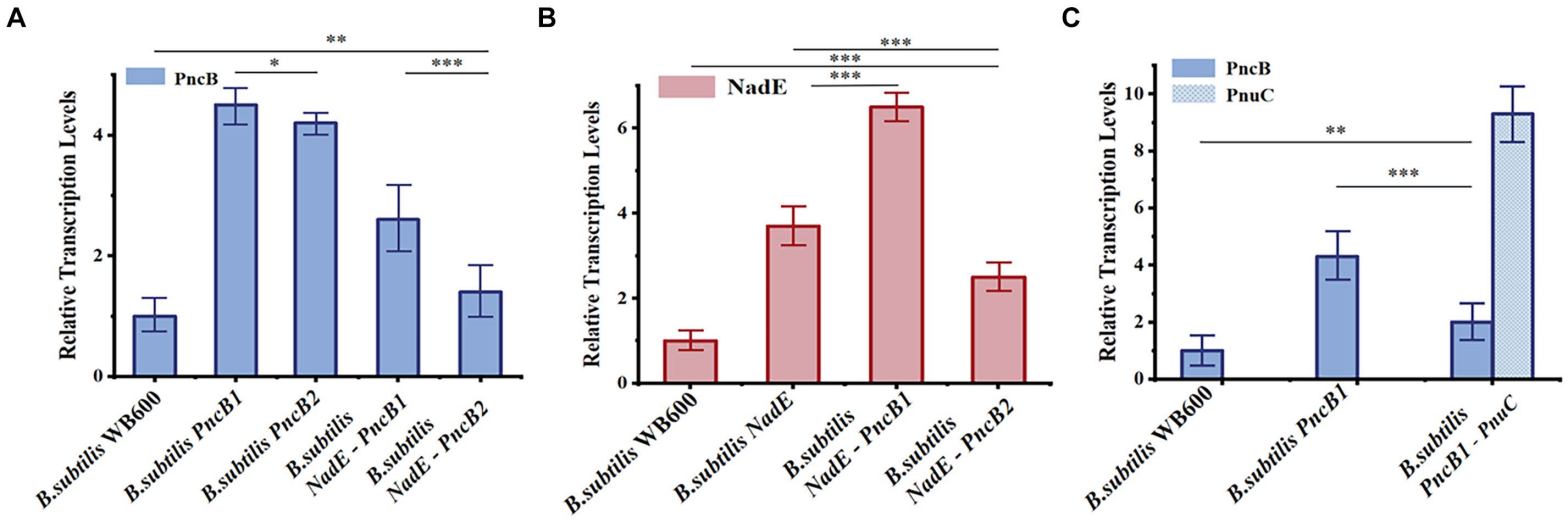
Relative transcriptional levels of NadE **(A)**, PncB **(B)**, and PnuC **(C)** in *B. subtilis* strains at 12 h. The mean and standard deviation of three independent experiments with biological replicates are shown using the values and error bars. Using a two-tailed *t*-test, *p < 0.05, **p < 0.01, and ***p < 0.001.

To study the effect of the transcriptional levels of *PncB*, *NadE*, and *PnuC* on NMN production, the expression of each gene was analyzed in recombinant and control strains of *B. subtilis* WB600, both of which were cultured under identical conditions for 12 h and analyzed via qRT-PCR. The results indicated that *PncB* transcriptional levels of the three engineered bacteria were 1.5–4.4-fold higher than those of the control strain (Figure 5A), with their NMN titers being increased to varying degrees (Figure 4A). These data support the hypothesis that *PncB* overexpression plays an important role in NMN biosynthesis. As shown in Figure 5A, the transcriptional level of *B. subtilis PncB1* was higher than that of *B. subtilis PncB2*, which is consistent with the higher NMN titer of *B. subtilis PncB1* (Figure 4A). This result showed that the number of terminators following MCS1 and MCS2 probably affects the gene expression intensity in *B. subtilis* (Figure 3). Furthermore, Lin et al. revealed that the tandem terminator can reinforce the heterologous gene expression (Lin et al., 2021a). The transcriptional levels of *NadE* in the three recombinant strains were significantly higher than those in the control *B. subtilis* WB600, indicating the successful construction of *NadE*-overexpressing recombinant strains (Figure 5B). However, the overexpression of *NadE* alone and coexpression with *PncB* did not significantly increase the NMN production of *B. subtilis NadE*, *B. subtilis NadE-PncB1*, and *B. subtilis NadE-PncB2* (Figure 4A). As shown in Figure 2, *NadE* is involved in two conversion pathways from NaMN to NMN (Foster and Moat, 1980; Begley et al., 2001). This may lead to the overexpression of *NadE*, which is not as effective as *PncB*.

*B. subtilis PncB1*-*PnuC* exhibited a significant increase in *PnuC* expression compared with *B. subtilis* WB600 and *B. subtilis PncB1* (Figure 5C). *PnuC* overexpression increased the extracellular NMN titer of *B. subtilis PncB1*-*PnuC* (Figure 4B). Overall, the results indicate that increasing the transcriptional levels of *PncB* and *PnuC* can efficiently enhance the production of NMN.

### 3.4 NMN production in a 5-L bioreactor

**Figure 6:**
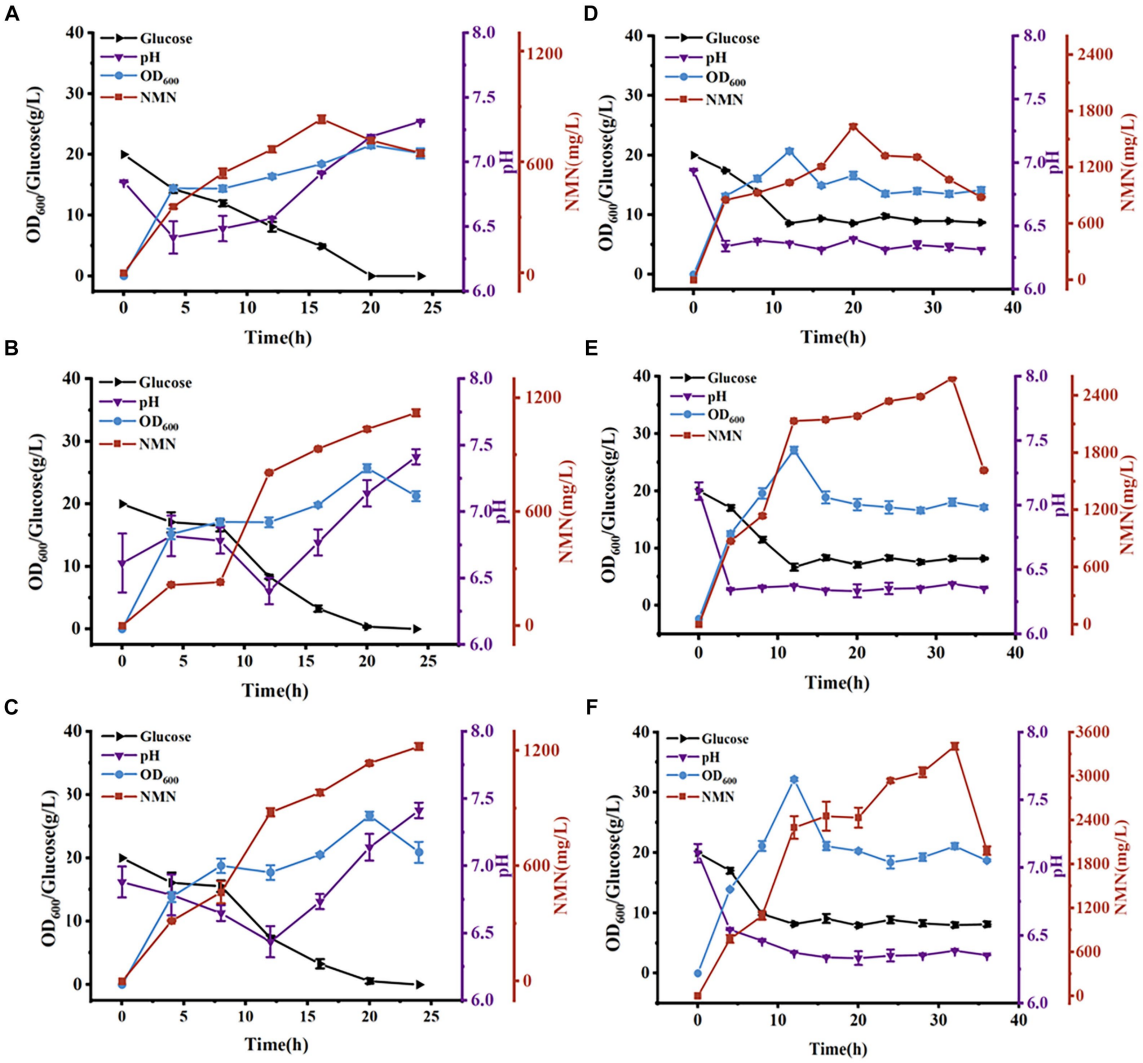
Time course of NMN production in a 5-L bioreactor. NMN production by *B. subtilis* WB600 **(A)**, *B. subtilis PncB1* **(B)**, and *B. subtilis PncB1-PnuC* **(C)** during batch fermentation. NMN production by *B. subtilis* WB600 **(D)**, *B. subtilis PncB1* **(E)**, and *B. subtilis PncB1-PnuC* **(F)** during fed-batch fermentation.

**Figure 7:**
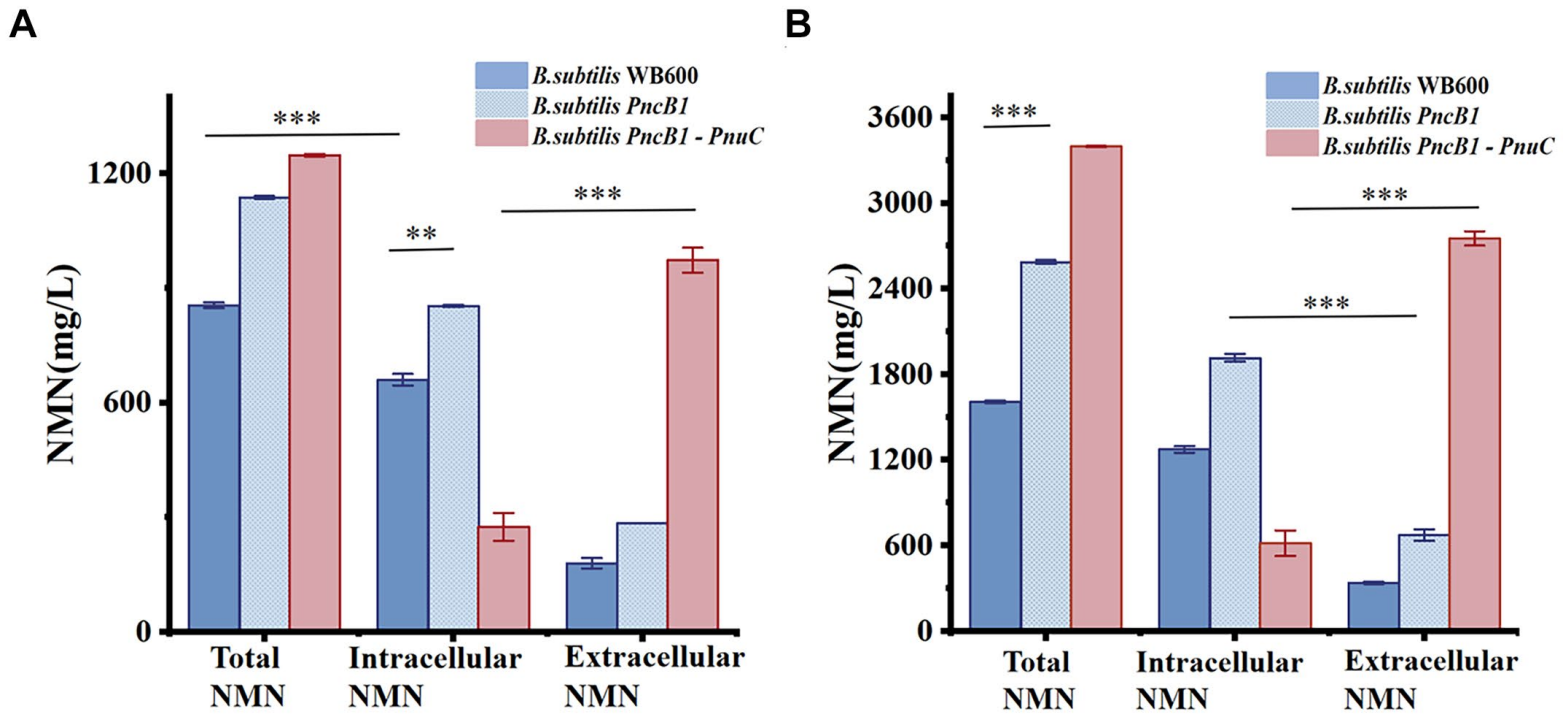
Effect of *PnuC* overexpression on NMN production during batch fermentation **(A)** and fed-batch fermentation **(B)** using a 5-L bioreactor. The mean and standard deviation of three independent experiments with biological replicates are shown using the values and error bars. Using a two-tailed *t*-test, *p < 0.05, **p < 0.01, and ***p < 0.001.

The total NMN titers of *B. subtilis PncB1* and *B. subtilis PncB1*-*PnuC* obtained via flask fermentation were similar. To further verify whether the functional activity of *PnuC* can improve the biosynthesis of NMN, batch fermentation and fed-batch fermentation strategies were employed. The engineered strains *B. subtilis PncB1* and *B. subtilis PncB1*-*PnuC* and the host strain *B. subtilis* WB600 were cultivated in a 5-L fermenter using the batch fermentation method. The pH, OD_600_, RG content, and NMN titer were measured during fermentation (Figures 6A–C). The total NMN titer of the control *B. subtilis* WB600 strain was 849.31 mg/L at 16 h, whereas the NMN titers of the two engineered strains reached 1,135 (24 h) and 1,245 (24 h) mg/L, respectively. The cell concentration (OD_600_) of the strains *B. subtilis* WB600, *B. subtilis PncB1*, and *B. subtilis PncB1-PnuC* reached 20.12, 25.66, and 26.32, respectively, after 20 h of batch culture (Figures 6A–C). NMN production in the engineered stains was slightly delayed by approximately 4 h compared with that in the control strain WB600, which corresponded to the overexpression of *PncB* and *PncB-PnuC*. After glucose consumption, pH initially decreased and then increased with the gradual increase in OD_600_ values. In the 5-L bioreactor, the extracellular NMN titer of *B. subtilis PncB1*-*PnuC* relative to the total NMN titer was 81.06%, whereas the titers of *B. subtilis* WB600 and *B. subtilis PncB1* reached only 21.17 and 25.32% of the total NMN titer, respectively (Figure 7A).

To further improve the NMN titer, the engineered strains were cultured in a 5-L fermenter via fed-batch fermentation. The OD_600_, pH, RG content, and NMN titer measured during fermentation are shown in Figures 6D–F. The control strain *B. subtilis* WB600 exhibited the highest NMN accumulation of 1,612 mg/L at 20 h. The NMN titer of the genetically engineered strain *B. subtilis PncB1* was 2,598 mg/L, whereas that of *B. subtilis PncB1-PnuC* reached a maximum of 3,396 mg/L at 32 h. Overexpression of *PncB1* in *B. subtilis PncB1* and co-overexpression of *PncB1* and *PnuC* in *B. subtilis PncB1-PnuC* resulted in an increase in the total NMN titer by 61.17 and 110.67%, respectively, compared with the host strain *B. subtilis* WB600. The extracellular NMN titer of *B. subtilis PncB1-PnuC* accounted for 76.96% of the total NMN titer, and the extracellular NMN titers of *B. subtilis* WB600 and *B. subtilis PncB1* accounted for only 19.57 and 23.12%, respectively, of the total NMN titer (Figure 7B). Under fed-batch fermentation conditions, the glucose consumption rate of the genetically engineered strains was slightly higher than that of the parental strain. The growth rates and biomass of *B. subtilis PncB1* and *B. subtilis PncB1-PnuC* were also higher than those of the parental strain. As shown in Figures 6D–F, the NMN titers of *B. subtilis PncB1* and *B. subtilis PncB1-PnuC* strains peaked at 36 h during fed-batch fermentation, which was delayed by 16 h compared with the WB600 strain (20 h). After entering the decay stage, as the bacterial cells autolyze, NMN production may suddenly and rapidly decrease. Consequently, it is necessary to terminate fermentation in a timely manner to obtain the optimal NMN production (Figure 6). In addition, the NMN production rate of *B. subtilis PncB1-PnuC* during fed-batch fermentation (106.12 mg/[L h]) increased by 30.72% compared with that during batch fermentation (81.18 mg/[L h]). Thus, the fed-batch fermentation strategy markedly increased the total and extracellular NMN titers of *B. subtilis PncB1-PnuC*.

## 4 Discussion

As one of the important precursors for producing NAD^+^, NMN has been widely recognized for its health and therapeutic effects in humans (Hosseini et al., 2019; Chen et al., 2020; Liu et al., 2021; Lin et al., 2021b). Chemical methods produce a small amount of physiologically inactive α-NMN (Walt et al., 1984); however, the high purification cost and nonreusability of enzymes in enzyme synthesis methods remain a problem for industrial production (Qian et al., 2022). Huang et al. obtained 16.2 g/L NMN in *E. coli* using nicotinamide as a precursor (Huang et al., 2022), which is currently the highest biosynthetic NMN titer. Therefore, the use of genetically engineered bacteria for large-scale fermentation to produce NMN has great potential, but the cost still needs to be further reduced. Compared with the abovementioned biological methods, our system does not require the preparation of purified enzymes, and NMN can be produced from inexpensive substrates and exported into the culture medium for easier extraction.

*B. subtilis* does not produce exotoxins and endotoxins; therefore, it is generally considered a safe strain that can utilize inexpensive carbon sources for large-scale fermentation (Liu et al., 2019; Zhang et al., 2021). Therefore, *B. subtilis* is more likely to fulfill the needs of industrial production as a cell factory. In the current study, we selected the NA pathway for metabolic modification to enhance the biosynthesis of NMN and successfully constructed six genetically engineered strains using *B. subtilis* WB600 as the chassis strain and pMA5 as the overexpression plasmid. All six genetically engineered strains increased the NMN titer via shake-flask fermentation compared with the parental WB600 strain. Following culture in a medium containing glucose and tryptone, the highest NMN titer of 3,396 mg/L was achieved via batch fermentation in a 5-L bioreactor, which was higher than the previously reported titer of 1.21 g/L in *B. subtilis* (Zhang et al., 2024).

Although the highest production of NMN from nicotinamide has been reported in *E. coli* (Huang et al., 2022), further studies are warranted to achieve more efficient production of NMN in *B. subtilis*. Furthermore, although the use of free plasmids for gene overexpression is effective, plasmids may be readily lost, leading to a decrease in stability during strain propagation. In the future, we will optimize the expression pathway, reinforce PRPP generation, increase the supply of ATP and precursors, and stabilize gene expression to further enhance the NMN titer.

## 5 Conclusion

Herein, we successfully overexpressed *PncB* and *NadE*, which can convert NA to NMN for higher NMN production. To further enhance the NMN titer, *PnuC* was synthesized and co-overexpressed with *PncB*. The total, extracellular, and intracellular NMN titers of the recombined strains were tested via flask fermentation, batch fermentation, and fed-batch fermentation in a medium containing glucose and peptone. The total NMN titer of the final *B. subtilis PncB1*-*PnuC* strain reached 3,396 mg/L at 36 h, with the highest productivity rate of 106.12 mg/[L h]. This was achieved via fed-batch fermentation using a 5-L bioreactor, which was superior to batch fermentation. Thus, we constructed a novel pathway for the *de novo* biosynthesis of NMN. Compared with the salvage pathway for NMN production from precursors such as NR or NAM in *E. coli* (Supplementary Table S4), our designed pathway for NMN production was more economical and safer because of the incorporation of inexpensive substrates and safe chassis strain.

## Data availability statement

The original contributions presented in the study are included in the article/Supplementary material, further inquiries can be directed to the corresponding author.

## Author contributions

ZT: Conceptualization, Funding acquisition, Methodology, Project administration, Resources, Supervision, Validation, Visualization, Writing – original draft, Writing – review & editing. YY: Data curation, Investigation, Software, Writing – original draft. YW: Data curation, Software, Investigation, Methodology, Writing – original draft. JY: Data curation, Investigation, Methodology, Writing – original draft. BZ: Data curation, Formal analysis, Software, Writing – original draft. YH: Writing – review & editing, Resources, Supervision. SJ: Writing – review & editing, Resources, Supervision.
