# Supplementary material for: Biosynthesis of β-nicotinamide mononucleotide from glucose via a new pathway in Bacillus subtilis
Source: Front Microbiol. 2024 Jun 11;15:1405736. doi: 10.3389/fmicb.2024.1405736 (PMC11197623; doi:10.3389/fmicb.2024.1405736)
Supplement: Supplementary file 1 [file Data_Sheet_1.pdf]

# *Supplementary Material*

## 1 Supplementary Tables

### Table 1. Strains and plasmids used in this study

| Strains and plasmids | Relevant genotype | Sources |
|---|---|---|
| plasmids | | |
| pMA5 | HpaII promoter, ColE1 Ori, F1 Ori | Tianjin Weike Biotech |
| pMA5-nadE | *NadE* in pMA5-MCS1, KanR | This study |
| pMA5-pncB1 | *PncB* in pMA5-MCS1, KanR | This study |
| pMA5-pncB2 | *PncB* in pMA5-MCS2, KanR | This study |
| pMA5-nadE-pncB1 | *NadE* and *PncB* in pMA5-MCS1, KanR | This study |
| pMA5-nadE-pncB2 | *NadE* in pMA5-MCS1, *PncB* in pMA5-MCS2, KanR | This study |
| pMA5-pncB-pnuC | *PncB* in pMA5-MCS1, *PnuC* in pMA5-MCS2, KanR | This study |
| Strains | | |
| *B. subtilis* 168 | Wild strain | HyunArt Bio-Tech |
| *B. subtilis* WB600 | Universal expression hosts | HyunArt Bio-Tech |
| *B. subtilis NadE* | *B. subtilis* WB600 with pMA5-nadE, KanR | This study |
| *B. subtilis PncB1* | *B. subtilis* WB600 with pMA5-pncB1, KanR | This study |
| *B. subtilis PncB2* | *B. subtilis* WB600 with pMA5-pncB2, KanR | This study |
| *B. subtilis NadE - PncB1* | *B. subtilis* WB600 with pMA5-nadE-pncB1, KanR | This study |
| *B. subtilis NadE - PncB2* | *B. subtilis* WB600 with pMA5-nadE-pncB2, KanR, | This study |
| *B. subtilis PncB1 - PnuC* | *B. subtilis* WB600 with pMA5-pncB-pnuC, KanR | This study |

**Table 2. Primers for construct expression vector**

| Primer | Sequence (5'-3') | Restriction site |
|---|---|---|
| Primers used to construct the pMA5-nadE plasmid | | |
| B. N/F | aaagtgaaatcagggggatccATGAGCATGCAGGAAAAGATTATG | *Bam*H I. |
| B. N/R | gagctcgactctagaggatccTTATTTCCACCAGTCATCAAACATAGA | |
| Primers used to construct the pMA5-pncB plasmid | | |
| B. P/F1 | aaaaggagcgatttacatatgGTGTTAGAGTACGGGATTTAAAGATGACA | *Nde*I |
| B. P/R1 | acaaactgcataactcatatgTTATTCTTCCTCAAGCTCTTCTTCAA | |
| B. P/F2 | cggtacctctagaagaagcttGTGTTAGAGTACGGATTTAAAGATGACA | *Hin*D III |
| B. P/R2 | ctttaccttgtctccaagcttTTTTATTCTTCCTTCAAGCTCTTCTTCAA | |
| Primers used to construct the pMA5-nadE-pncB plasmid | | |
| pNP/F1 | aaaaggagcgatttacatatgGTGGAGATTTTTTGAGTGATCTTCTC | *Nde* I |
| pNP/R1 | acaaactgcataactcatatgTTATTCTTCCTCAAGCTCTTCTTCAA | |
| pNP/F2 | cggtacctctagaagaagcttGTGTTAGAGTACGGATTTAAAGATGACA | *Hin*D III |
| pNP/R2 | tctggtacgtaccaagctagcCTTTTTGCATTCTACAAACTGCATAA<br>ctttaccttgtctccaagcttTTTTATTCTTCCTTCAAGCTCTTCTTCAA | |
| Primers used to construct thepMA5-pncB-pnuC plasmid | | |
| pPP/F | cggtacctctagaagaagcttATGGTTAGAAGTCCACTTTTTTTTTACTCA | *Hin*D III |
| pPP/R | ctttaccttgtctccaagcttCTAAATATAATTATTTACACGTTCTCGCTC | |

## Table 3. Primers used for qRT-PCR

| Primer | Sequence (5'-3') | Amplicon size (bp) |
|---|---|---|
| Primers used to verify the pMA5-nadE plasmid | | |
| nadE-F1 | ggatcttccagagatATTGGCAAGGGTTTAAAGGTGG | 1356 |
| nadE-R1 | ctgccgttcgacgatAATAATAATTTTTTCACGTTGAAAATCTC | |
| Primers used to verify the pMA5-pncB plasmid | | |
| pncB-F1 | ggatcttccagagatTTCTCAAAAAATACTACCTGTCCCTTG | 1703 |
| pncB-R1 | ctgccgttcgacgatGCATAACTCATATGTTATTCTTCCTCAA | |
| pncB-F2 | ggatcttccagagatTGGGATATATCAACGGTGGTATATCC | 2004 |
| pncB-R2 | ctgccgttcgacgatAGATAAGAAAGAACAAGTTCAAAACCA | |
| Primers used to verify the pMA5-nadE-pncB plasmid | | |
| np-F1 | ggatcttccagagatAGACAAAACGGACAAAATAAAAATTG | 3091 |
| np-R1 | ctgccgttcgacgatTCGACCTCTAGAACGCGTGATC | |
| Primers used to verify thepMA5-pncB-pnuC plasmid | | |
| npp-F | ggatcttccagagatCATATGAGTTATGCAGTTTGTAGAATGC | 1338 |
| npp-R | ctgccgttcgacgatTAAATCGCTCCTTTTTAGGTGGC | |

**Table 4. Comparison of NMN production cost using different biosynthetic methods**

| Substrate | Huang Z et al. [25] | Shoji S et al. [23] | This study |
|---|---|---|---|
| NAM (g/L) | 8.3 | 7 | — |
| Glucose (g/L) | ≥ 27 | 46.6 | 45 |
| Yeast Extract (g/L) | 10 | 48 | 24 |
| Tryptone (g/L) | — | — | 12 |
| IPTG (g/L) | 0.119 | 0.023 | — |
| Tryptophan (g/L) | — | 38.4 | — |
| Others (g/L) | 29.658 | 10 | 14.84 |
| NMN Yield (g/L) | 16.2 | 6.79 | 3.39 |
| Culture medium cost （$ /Kg NMN） | 16.44 | 554.47 | 52.35 |
| Conversion rate (%) (g Glucose/g NMN) | — | — | 7.5 |
| Molar conversion rate (%) (mol NAM/mol NMN) | 97 | 35 | — |

## 2 Supplementary Figures

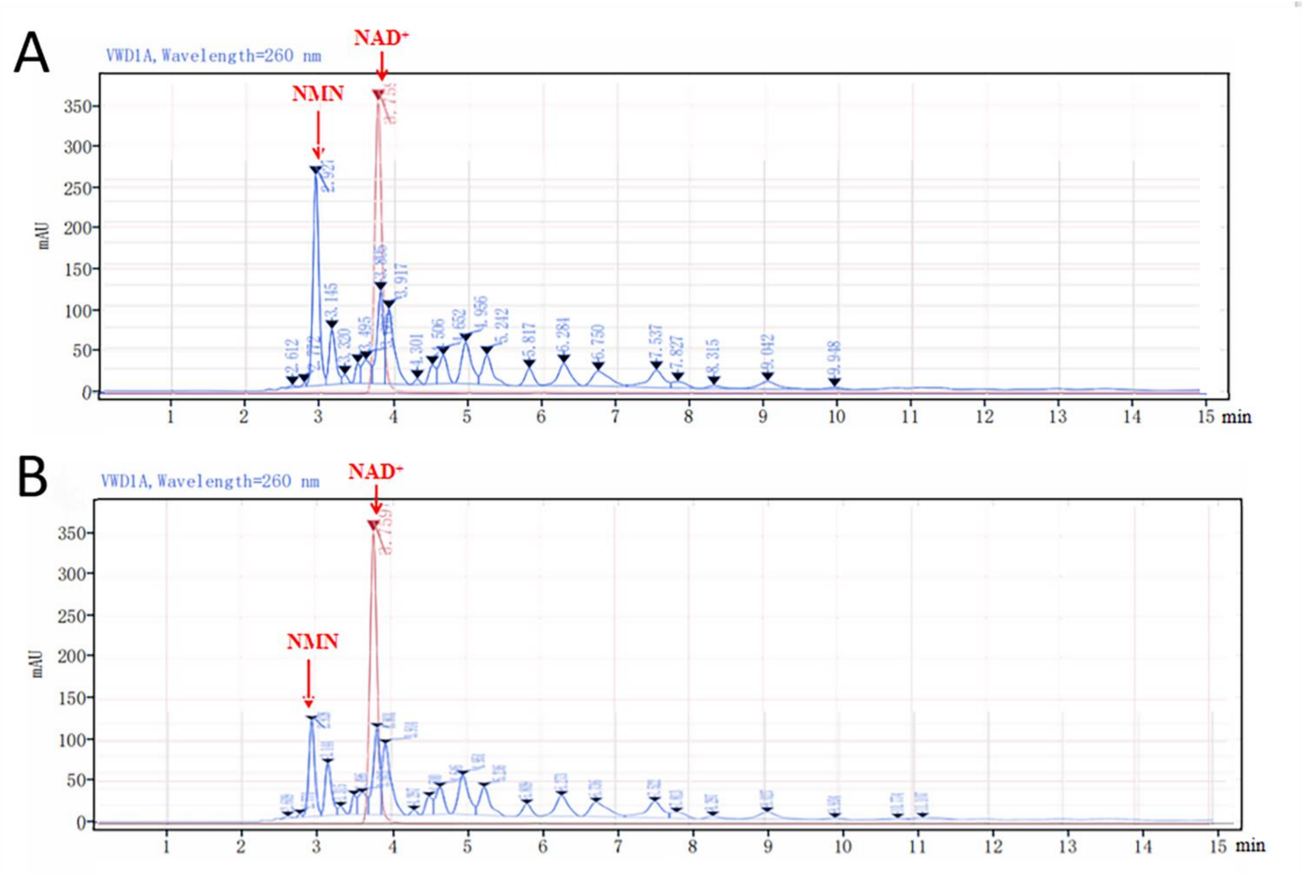


**Supplementary Figure 1.** HPLC chromatogram of $NAD^+$ in fermentation broth of different strains

Note: **(A)** $NAD^+$ content of *B. subtilis PncB1* (diluted 5 times) after 12 hours of fermentation in shake-flask. **(B)** $NAD^+$ content of *B. subtilis WB600* (diluted 5 times) after 12 hours of fermentation in shake-flask. Red line: 200 mg/L $NAD^+$ standard sample.

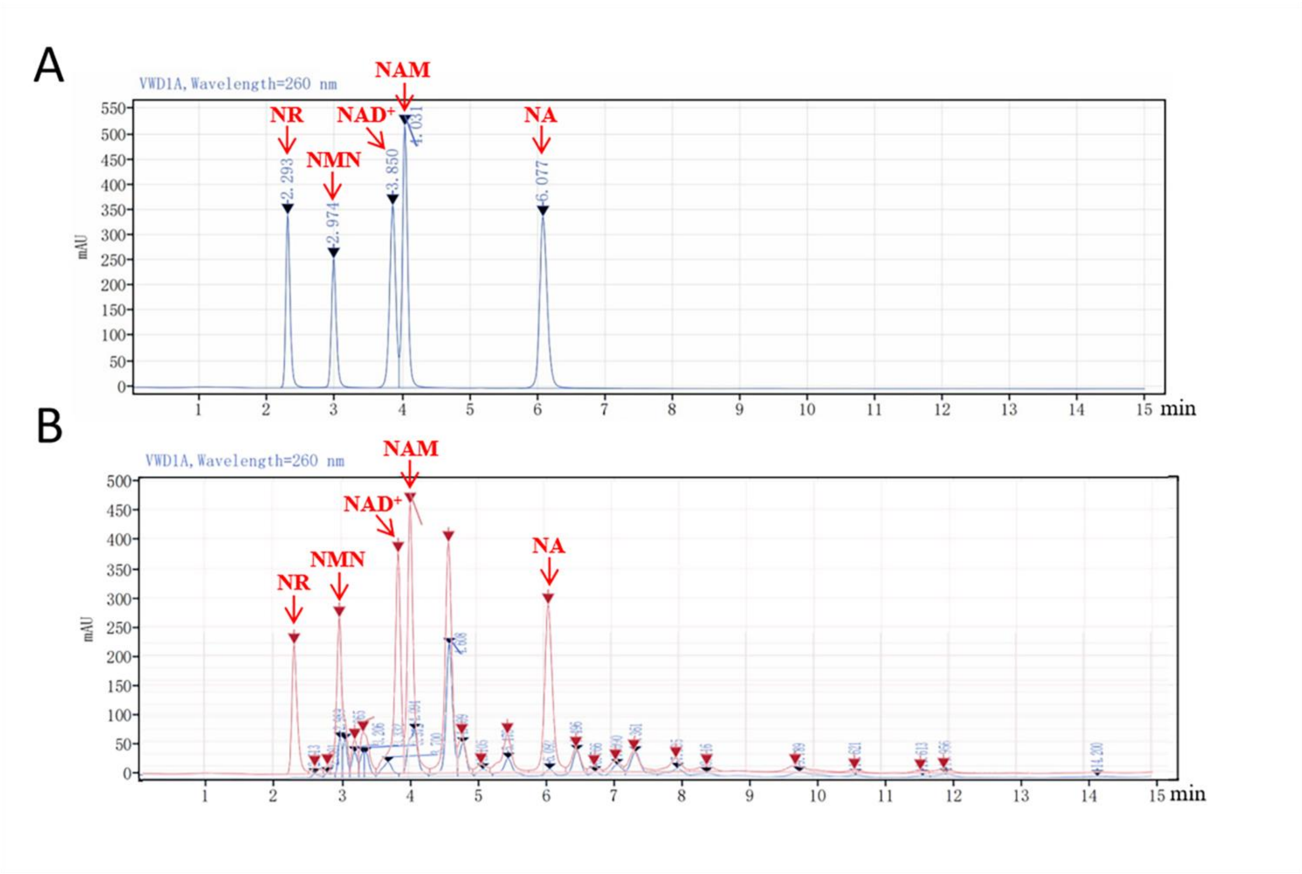


**Supplementary Figure 2.** Contents of NMN, NR, $NAD^+$, NAM and NA in the medium.

Note: **(A)** HPLC chromatogram of the mixture of NMN, NR, $NAD^+$, NAM and NA standard samples (200mg/L). **(B)** HPLC chromatogram of blank fermentation medium (diluted 5 times, blue line) and blank fermentation medium added the NMN, NR, $NAD^+$, NAM and NA standard samples (red line,160mg/L).
